# Influence of pelvic floor disorders on quality of life in women

**DOI:** 10.3389/fpubh.2023.1180907

**Published:** 2023-10-24

**Authors:** Rocío Adriana Peinado Molina, Antonio Hernández Martínez, Sergio Martínez Vázquez, Juan Miguel Martínez Galiano

**Affiliations:** ^1^Department of Nursing, University of Jaén, Jaén, Spain; ^2^Department of Nursing, Physiotherapy and Occupational Therapy, Faculty of Nursing, University of Castilla la Mancha, Ciudad Real, Spain; ^3^Epidemiology and Public Health CIBER (CIBERESP), Madrid, Spain

**Keywords:** pelvic floor disorders, quality of life, women's health, pelvic floor, women's health services

## Abstract

**Objective:**

To determine whether the different pelvic floor disorders are associated with changes in perceived quality of life (QoL), globally and in its sub-dimensions.

**Methods:**

An observational study was conducted with women in Spain between 2021 and 2022. Information was collected using a self-developed questionnaire on sociodemographic data, employment, history and health status, lifestyle and habits, obstetric history, and health problems. The SF-12 questionnaire was used to assess quality of life. The Pelvic Floor Distress Inventory (PFDI-20) was used to assess the presence and impact of pelvic floor problems, and includes the POPDI-6 subscales for prolapse, CRADI- 8 for colorectal symptoms, and UDI-6 for urinary symptoms. Crude (MD) and adjusted mean differences (aMD) were estimated with their respective 95% confidence intervals (CI).

**Results:**

Thousand four hundred and forty six women participated in the study with a mean age of 44.27 (SD = 14.68). A statistical association was observed between all the pelvic floor disorders and QoL, overall and in all its dimensions (*p* <*0*.001), in the bivariable analysis. The lowest scores were observed in the emotional component. After adjusting for confounding factors, the pelvic floor disorders in general (aMD −0.21, 95% CI: −0.23 to −0.20), the impact of uterine prolapse symptoms (aMD −0.20, 95% CI: −0.27 to −0.12), the colorectal-anal symptoms (aMD −0.15, 95% CI: −0.22 to −0.09), and urinary symptoms (aMD −0.07, 95% CI: −0.13 to −0.03) was negatively associated on the score on the SF-12 questionnaire (*p* <*0*.05).

**Conclusions:**

Women who have a pelvic floor dysfunction, symptoms of pelvic organ prolapse, colorectal-anal symptoms, or urinary symptoms, have a worse perceived quality of life in all dimensions. Prolapse symptoms have the biggest impact, and the emotional component of QoL is the most affected sub-domains.

## Introduction

Pelvic floor disorders are a public health problem, with their prevalence reaching 25% in the US in healthy non-pregnant women. It is a problem present in all age ranges, although occurs more in older women (1, 2). Hence, it is estimated that one in nine women will require surgical intervention for this problem at some point in their life. Pelvic floor disorders encompass clinical disorders related to urinary incontinence, pelvic organ prolapse, fecal incontinence, and pelvic-perineal pain syndrome (2–4).

Recent studies have identified that the presence of these pelvic floor disorders and the impact of their symptoms reduce health-related quality of life (HRQoL) in women; likewise, they have direct negative consequences on physical, psychological, sexual, and social health (5–16). These disabling problems lead to social isolation, affect the performance of tasks, cause loss of personal and intimate relationships, and reduce participation in leisure activities (17).

The World Health Organization (WHO) defines “*Quality of Life as an individual's perception of their position in life in the context of the culture and value systems in which they live and in relation to their goals, expectations, standards and concerns*” (18). HRQoL has become a fundamental indicator when evaluating health care and reflects a person's physical, psychological, social, and emotional wellbeing (19, 20). Measuring HRQoL is a challenge for researchers as it is a subjective, individual term that evolves over time (20). However, a low HRQoL score can be used to predict physical deterioration, resource consumption rates, hospital admissions, and even mortality (21, 22). There is evidence that women report lower HRQoL scores than men (23–27).

The scores obtained in previous HRQoL research differ depending on sociodemographic, socioeconomic, and gender characteristics, among others (25, 28). In addition, some studies have shown that a low HRQoL score has been associated with modifiable factors such as lifestyle (29), physical exercise (30), being overweight (31), sleep quality (32), among others.

The impact of pelvic floor disorders and their symptoms on HRQoL in women has been studied with inconclusive results; however, not all disorders have been considered, and most of the studies were carried out around the perinatal stage, ignoring other stages of a woman's life (33, 34). The prevalence of these disorders demonstrates their presence in a high number of women. As such, the repercussion on health and other aspects such as quality of life is substantial and pushes it to the frontline as a public health and women's health problem that needs to be addressed (13–16). For this reason, the objective of the present study is to determine whether the different pelvic floor disorders are associated with changes in perceived quality of life, globally and in its sub-dimensions, with the aim that professionals can take these into account in their clinical practice and prioritize the most appropriate interventions.

## Materials and methods

### Design and subject selection

This observational study was carried out during 2021 and 2022 in Spain. Exclusion criteria were: difficulty understanding Spanish, women under 18 years of age, having a mental and/or cognitive disorder that may affect data collection, and having given birth in the last 12 months or were pregnant.

To carry out this study it was necessary to recruit a minimum sample of 890 women based on the following criteria: a confidence level of 95%, an absolute precision error of 3%, a population prevalence of pelvic floor problems of around 25% (1), and a percentage of losses of 10%.

### Information sources and study variables

The investigation was publicized by places that women frequented: women's associations, information centers as well as health centers. When the women expressed their interest in participating, their informed consent was obtained, after which trained observers interviewed them to obtain sociodemographic and employment data, previous medical history and health status, lifestyle and habits, obstetric history, and health problems using a previously piloted self-made questionnaire. The women were recruited consecutively,

Next, the women's quality of life was assessed using the SF-12 questionnaire (35). This questionnaire consists of a set of 12 items on HRQoL, and considers physical functioning through 6 items and, another 6 items to assess the state of perceived mental health. The SF-12 version presents eight domains: physical functioning, role physical, bodily pain, general health, vitality, social functioning, role emotional, and mental health. The total quality of life score is obtained from the sum of the two subscales, physical health, and mental health, on a scale from 0 to 100, with a higher score indicating a better perceived HRQoL. The value 50 (SD = 10) is the general population's mean values, higher or lower than this are interpreted as better or worse quality of life, respectively (35).

Next, to assess the presence and impact of pelvic floor disorders, the Pelvic Floor Distress Inventory (PFDI-20) was used (36, 37). The PFDI-20 includes 20 items divided into 3 symptom scales: symptoms of pelvic organ prolapse (POPDI-6) (questions 1 to 6); colorectal-anal symptoms (CRADI-8) (questions 7–14); and urinary symptoms (UDI-6) (questions 15–20). To determine the prevalence of the different disorders, the following key questions/items were used: Regarding the prevalence of prolapse, the criterion of symptomatic prolapse was used by means of an affirmative answer in item 3; for fecal incontinence, the sum of affirmative answers in items 9 and 10 was used as a criterion; for urinary incontinence, the sum of affirmative responses to items 16 and 17; while for the prevalence of pelvic pain, the affirmative response to item 20 was used. Each question used a 0–4 response format, categorizing the dysfunction in 4 levels: none, little, moderate, a lot. The minimum score for each subscale is 0, and the maximum is 100 points, referring to minimum and maximum dysfunction. The total score of the PFDI-20 is the sum of the three subscales, with a maximum score of 300.

Next, the short version of the International Physical Activity Questionnaire (IPAQ) was used to determine the level of physical activity (38). The IPAQ classifies adult populations based on activity levels (low, moderate, and high). This questionnaire has adequate validity. A low level of physical or inactive activity was considered when 600 METs (Physical Activity Unit of the Test) were not reached.

Finally, to assess the quality of sleep, the validated Pittsburgh Sleep Quality Index (PSQI) (39) was used, which comprises 19 self-assessed questions. Based on these questions, seven components were elaborated that evaluated different aspects of sleep quality: subjective sleep quality, sleep latency, sleep time, total sleep efficiency, sleep disorders, consumption of hypnotic drugs, and daytime dysfunction. The scores for each question range from 0 to 3 points, with 0 corresponding to the absence of a problem, and 3 with a severe sleep problem. Finally, to determine the scale's total score, the scores of these components are summed, resulting in a minimum score of 0 points and a maximum of 21 points. Participants with a total score of 0 to 4 were considered to have good sleep quality, and scores equal to or >5 are interpreted as poor sleep quality (40).

### Statistical analysis

SPSS 28.0 was used for the analysis of the information. First, descriptive statistics were carried out using absolute and relative frequencies, and means with standard deviation (SD) for continuous variables.

Next, a bivariable analysis was performed between the pelvic floor disorders (urinary and fecal incontinence, prolapse, and pain) and the sub-dimensions of the SF-12 quality of life questionnaire using the Student-Fisher *t*-test. In addition, the relationship between the impact of symptoms through the PFDI-20 scale and its subscales POPDI-6, CRADI-8, and UDI-6 with the different dimensions of the SF-12 questionnaire was analyzed by means of linear regression.

Finally, we performed bivariable and multivariable analyses using multiple linear regression between the three subscales of the impact of pelvic floor dysfunction symptoms and quality of life. To do this, we adjusted for age, physical activity, sleep quality, the existence of menopause, body mass index (BMI), and the presence of different comorbidities. In all cases, mean differences (MD) with their respective 95% confidence intervals (95% CI) were estimated.

### Ethical considerations

The study received a favorable opinion from the Research Ethics Committee of the province Jaén, reference number SPCV-0220/0302-N-20. Before starting the questionnaire, the women had to read an information sheet about the study and its objectives and confirm their consent to participate in it.

## Results

A total of 1446 women participated. Their mean age was 44.27 years (SD = 14.68), with a mean BMI of 25.00 (SD = 4.75). Regarding civil status, 57.3% (828) were married. In terms of lifestyle, 14.3% (207) of the sample smoked, 54.4% (786) drank alcohol occasionally, and 35.4% (512) had a median income level of 1,000–1,999 euros.

Regarding personal and obstetric history, 28.9% (418) were in menopause, 33.0% (477) had some type of illness, of which 6.4% (93) were musculoskeletal conditions. Urinary incontinence was present in 55.8% (807) of participants, and 18.7% (271) had pelvic pain. In addition, 78.2% (1131) had been pregnant at least once. Regarding the type of delivery, 67.2% (971) had experienced a vaginal birth and 26.2% (379) an instrumental one (Table 1).

**Table 1:** Sociodemographic and clinical characteristics of the study sample.

**Variable**	***n* (%)**	**Mean (SD)**
**Age**		44.27 (14.68)
< 30 years	220 (15.2)	
30–49.9 years	799 (55.3)	
≥50	427 (29.5)	
**BMI**		25.00 (4.75)
Normal weight < 24.99	830 (57.4)	
Overweight 25–29.9	405 (28.0)	
Obesity ≥ 30	211 (14.6)	
**Civil status**		
Single	334 (23.1)	
Separated	19 (1.3)	
Divorced	73 (5.0)	
Widowed	67 (4.6)	
Common-law couple	125 (8.6)	
Married	828 (57.3)	
**Employment sector**		
Administration	126 (8.7)	
Agriculture/livestock	29 (2.0)	
Commerce	59 (4.1)	
Student	105 (7.3)	
Industry and construction	54 (3.7)	
Retired	165 (11.4)	
Self-employed	168 (11.6)	
Public servant	740 (51.2)	
**Income level**		
< 1,000 euros	196 (13.6)	
1,000–1,999 euros	512 (35.4)	
2,000–2,999 euros	425 (29.4)	
>3,000 euros	313 (21.6)	
**Alcohol consumption**		
Never	351 (24.3)	
Occasionally	786 (54.4)	
Only weekends	144 (10.0)	
Frequently	142 (9.8)	
Daily	23 (1.6)	
**Smoking habit**		
No	1239 (85.7)	
Yes	207 (14.3)	
**Pregnancy**		
None	315 (21.8)	
One	194 (13.4)	
Two or more	937 (64.8)	
**Vaginal birth**		
None	475 (32.8)	
One	289 (20.0)	
Two or more	682 (47.2)	
**Instrumental birth**		
No	1067 (73.8)	
Yes	379 (26.2)	
**Tear**		
No	904 (62.5)	
Yes	542 (37.5)	
**Menopause**		
No	1028 (71.1)	
Yes	418 (28.9)	
**Illness**		
No	969 (67.0)	
Yes	477 (33.0)	
**Cardiovascular disorder**		
No	1244 (86.0)	
Yes	123 (8.5)	
**Respiratory disorder**		
No	1329 (91.9)	
Yes	38 (2.6)	
**Endocrine disorder**		
No	1219 (84.3)	
Yes	148 (10.2)	
**Gynecological disorder**		
No	1325 (91.6)	
Yes	42 (2.9)	
**Musculoskeletal disorder**		
No	1274 (88.1)	
Yes	93 (6.4)	
**Neurological disorder**		
No	1333 (92.2)	
Yes	34 (2.4)	
**Neoplastic disease**		
No	1355 (93.7)	
Yes	12 (0.8)	
**Gastrointestinal disorder**		
No	1325 (91.6)	
Yes	42 (2.9)	
**Dermatological disorder**		
No	1345 (93.0)	
Yes	22 (1.5)	
**Mental illness**		
No	1342 (92.8)	
Yes	25 (1.7)	
**Nephro-urological disorder**		
No	1357 (93.8)	
Yes	10 (0.7)	
**Immunological disorder**		
No	1356 (93.8)	
Yes	11 (0.8)	
**Ophthalmo-ENT disorder**		
No	1342 (92.8)	
Yes	25 (1.7)	
**Urinary incontinence**		
No	639 (44.2)	
Yes	807 (55.8)	
**Fecal incontinence**		
No	1296 (89.6)	
Yes	150 (10.4)	
**Prolapse**		
No	1243 (86.0)	
Yes	203 (14.0)	
**Pelvic pain**		
No	1175 (81.3)	
Yes	271 (18.7)	

Table 2 shows the response distribution of HRQoL according to the SF-12 questionnaire, as well as the dimensions that evaluated the domains of physical and mental health. Of note, 50.9% (736) of the women reported alterations in the vitality domain, which was the most affected dimension with a mean score of 52.72 (SD = 23.49). In addition, 15.3% (221) of the women rated their perceived general health as low, with a mean score of 55.43 (SD = 19.28). In general, in the total SF-12, 16.5% (239) of the participating women presented altered HRQoL, with a mean score of 69.51 (SD = 20.09).

**Table 2:** Distribution of responses to the HRQoL questionnaire SF-12.

**Dimension scores SF-12**	***n* (%)**	**Mean (SD)**
**Physical function**		85.29 (25.91)
>50	1350 (93.4)	
< 50	96 (6.6)	
**Physical role**		71.85 (40.83)
>50	1142 (79.0)	
< 50	304 (21.0)	
**Body pain**		80.53 (25.82)
>50	1311 (90.7)	
< 50	135 (9.3)	
**Vitality**		52.72 (23.49)
>50	710 (49.1)	
< 50	736 (50.9)	
**Social function**		75.05 (25.56)
>50	1210 (83.7)	
< 50	236 (16.3)	
**Emotional role**		60.55 (46.00)
>50	955 (66.0)	
< 50	491 (34.0)	
**Mental health**		74.63 (16.96)
>50	1351 (93.4)	
< 50	95 (6.6)	
**General health**		55.43 (19.28)
>50	1225 (84.7)	
< 50	221 (15.3)	
**SF-12 total**		69.51 (20.09)
>50	1207 (83.5)	
< 50	239 (16.5)	

Next, the bivariable analysis in Table 3 shows the relationship between pelvic floor disorders and HRQoL using the SF-12 questionnaire. This analysis observed a significant association in all cases (*p* <*0*.05). Specifically, regarding women who have urinary incontinence, the most affected dimension was vitality, with a mean score of 50.68 (SD = 23.16). Whereas, for women who have fecal incontinence, the most affected domain was the emotional role, with a mean score of 40.67 (SD = 45.39). Regarding women with uterine prolapse, the most affected dimension was vitality, showing a mean score of 45.02 (SD = 23.64). For women with pelvic pain, the most altered domain was the emotional role, with a score of 40.59 (SD = 46.09). Finally, as shown by the scores in the table, it was reported that the mental component was always the most affected in all pelvic floor disorders.

**Table 3:** SF-12 Quality of life questionnaire and the sub-dimensions related to pelvic floor dysfunction. Bivariate analysis.

**SF-12 dimensions**		**Urinary incontinence**			**Fecal incontinence**			**Prolapse**			**Pelvic pain**		
		**No (*****n*** = **639)**	**Yes (*****n*** = **807)**	**Sig**.	**No (*****n*** = **1296)**	**Yes (*****n*** = **150)**	**Sig**.	**No (*****n*** = **1243)**	**Yes (*****n*** = **203)**	**Sig**.	**No (*****n*** = **1175)**	**Yes (*****n*** = **271)**	**Sig**.
Physical function	Mean (SD)	90.73 (20.07)	80.98 (29.03)	**< 0.001**	87.77 (22.53)	63.83 (39.77)	**< 0.001**	87.21 (24.02)	73.52 (33.06)	**< 0.001**	88.45 (22.77)	71.59 (33.26)	**< 0.001**
Physical role	Mean (SD)	79.10 (35.82)	66.11 (43.58)	**< 0.001**	74.85 (39.05)	46.00 (46.53)	**< 0.001**	74.62 (39.28)	54.93 (45.87)	**< 0.001**	77.66 (37.61)	46.68 (44.59)	**< 0.001**
Body pain	Mean (SD)	86.66 (21.44)	75.68 (27.89)	**< 0.001**	82.64 (24.22)	62.33 (31.56)	**< 0.001**	82.18 (24.86)	70.44 (29.13)	**< 0.001**	83.98 (23.73)	65.59 (29.01)	**< 0.001**
Vitality	Mean (SD)	55.31 (23.66)	50.68 (23.16)	**< 0.001**	53.78 (23.26)	43.60 (23.52)	**< 0.001**	53.98 (23.23)	45.02 (23.64)	**< 0.001**	55.30 (23.20)	41.55 (21.41)	**< 0.001**
Social function	Mean (SD)	77.65 (24.46)	72.99 (26.22)	**0.001**	76.77 (24.36)	60.13 (30.43)	**< 0.001**	76.12 (24.89)	68.47 (28.48)	**< 0.001**	77.99 (24.20)	62.29 (27.36)	**< 0.001**
Emotional role	Mean (SD)	64.32 (44.91)	57.56 (46.65)	**0.005**	62.85 (45.52)	40.67 (45.39)	**< 0.001**	62.71 (45.39)	47.29 (47.51)	**< 0.001**	65.15 (44.74)	40.59 (46.09)	**< 0.001**
Mental health	Mean (SD)	77.73 (14.93)	72.18 (18.04)	**< 0.001**	75.94 (15.93)	63.33 (20.97)	**< 0.001**	75.66 (16.43)	68.37 (18.74)	**< 0.001**	77.12 (15.60)	63.84 (18.36)	**< 0.001**
General health	Mean (SD)	59.12 (18.09)	52.51 (19.71)	**< 0.001**	56.71 (18.83)	44.33 (19.68)	**< 0.001**	56.32 (18.83)	50.00 (21.11)	**< 0.001**	72.91 (17.88)	54.76 (22.41)	**< 0.001**
SF-12 total	Mean (SD)	73.73 (17.15)	66.09 (21.55)	**< 0.001**	71.41 (18.65)	53.03 (24.23)	**< 0.001**	71.10 (19.12)	59.76 (22.99)	**< 0.001**	72.90 (17.88)	54.76 (22.41)	**< 0.001**

Bold numerical values are statistically significant values.

Next, Table 4 shows the analysis of the relationship between the impact of pelvic floor symptoms and perceived quality of life, with a statistical association observed with all four scales (*p* < 0.05). Overall, it was concluded that for each point of variation of the PFDI scale there is a reduction of 0.21 points in the SF-12 quality of life questionnaire, MD −0.21, 95% CI: −0.23 to −0.20. Of note, the symptoms of prolapse are the ones that most affect the assessment of quality of life, causing a decrease of 0.57 points in the total score of the SF-12 for each point of variation in the POPDI-6 subscale, MD −0.57, 95% CI: −0.62 to −0.51.

**Table 4:** Relationship between the impact of pelvic floor symptoms and quality of life.

**Variable**	**SF-12 total**	
**Score impact of pelvic floor problems**	**MD (95% CI)**	***P*** **-value**
Prolapse symptoms (POPDI-6)	−0.57 (−0.62 to −0.51)	**< 0.001**
Colorectal-Anal symptoms(CRADI-8)	−0.54 (−0.60 to −0.49)	**< 0.001**
Urinary symptoms (UDI-6)	−0.39 (−0.43 to −0.34)	**< 0.001**
Pelvic function disorders Total (PFDI-20)	−0.21 (−0.23 to −0.20)	**< 0.001**

Bold numerical values are statistically significant values.

Finally, a multivariable analysis was carried out, as shown in Table 5. In this analysis, the different sociodemographic variables, lifestyles, obstetric variables, personal history, and the different pelvic floor disorders and their impact were related to the total SF-12 questionnaire score. Being aged between 30 and 50 years and the performance of physical exercise, as evaluated by the IPAQ questionnaire, produced an improvement in the result of the total SF-12 with a MD of 4.82 points, 95% CI: 2.44–7.20 and 0.001 points, 95% CI: 0.001–0.001, respectively. On the other hand, when the BMI increased, the SF-12 score decreased, with a MD of −0.36 points, 95% CI: −0.54 to −0.17. Likewise, it was reported that women who had certain conditions had reduced HRQoL, namely musculoskeletal, MD −9.32, 95% CI: −13.03 to −5.62, neoplastic diseases MD −13.99, 95% CI: −23.10 to −4.88, and mental health illnesses MD −8.47, 95% CI: −14.70 to −2.42. Finally, the intensity of the impact of the symptoms of pelvic floor disorders, as assessed by the subscales, significantly decreased HRQoL: POPDI-6: MD −0.20, 95% CI: −0.27 to −0.12, CRADI-8: MD −0.15, 95% CI: −0.22 to −0.09 and UDI-6: MD −0.07, 95% CI: −0.13 to −0.03.

**Table 5:** Variable and multivariate analysis. Quality of life and associated factors.

	**Bivariable analysis SF-12 total**		**Multivariable analysis SF-12 total**	
**Variable**	**MD (95% CI)**	**P-value**	**MD (95% CI)**	**P-value**
Age 30–50 years (Ref Cat < 30 years)	2.27 (–0.68 to 5.22)	0.132	4.82 (2.44 to 7.20)	**< 0.001**
Age ≥ 50 years. (Ref Cat < 30 years)	−6.15 (–9.36 to−2.93)	**< 0.001**	3.37 (−0.92 to 7.67)	0.123
BMI (for each point)	−1.02 (−1.23 to −0.80)	**< 0.001**	−0.36 (−0.54 to −0.17)	**< 0.001**
Menopause (Ref Cat No)	−6.87 (−9.18 to −4.56)	**< 0.001**	−2.49 (−5.79 to 0.82)	0.140
Cardiovascular condition (Ref Cat No)	−16.86 (–20.50 to −13.22)	**< 0.001**	−3.21 (−6.56 to 0.13)	0.060
Pulmonary condition (Ref Cat No)	−10.77 (−17.28 to −4.25)	**0.001**	−1.67 (−6.71 to 3.37)	0.515
Endocrine condition (Ref Cat No)	−8.53 (−11.94 to −5.12)	**< 0.001**	−2.49 (−5.15 to 0.180)	0.068
Gynecology condition (Ref Cat No)	−9.06 (−15.34 to −2.78)	**0.005**	−4.11 (−8.98 to 0.76)	0.098
Musculoskeletal condition (Ref Cat No)	−23.95 (−27.99 to −19.92)	**< 0.001**	−9.32 (−13.03 to −5.62)	**< 0.001**
Neurological condition (Ref Cat No)	−17.13 (−23.88 to −10.38)	**< 0.001**	−5.28 (−10.62 to −0.059)	0.053
Neoplastic condition (Ref Cat No)	−22.94 (−34.75 to −11.13)	**< 0.001**	−13.99 (−23.10 to −4.88)	**0.003**
Gastrointestinal condition (Ref Cat No)	−13.66 (−19.84 to −7.48)	**< 0.001**	−2.48 (−7.26 to 2.31)	0.311
Dermatological condition (Ref Cat No)	−4.16 (−12.59 to 4.27)	0.334	−1.42 (−7.84 to 5.00)	0.664
Mental health disorder (Ref Cat No)	−25.78 (−33.58 to −17.98)	**< 0.001**	−8.47 (−14.70 to −2.42)	**0.008**
Nephro-urological condition (Ref Cat No)	−11.33 (−23.76 to 1.11)	0.074	8.10 (−1.68 to 17.87)	0.105
Immunological condition (Ref Cat No)	−8.12 (−20.56 to 4.33)	0.201	−6.46 (−15.91 to 3.00)	0.180
Ophthalmological-ENT condition (Ref Cat No)	−20.17 (−28.35 to −12.00)	**< 0.001**	−2.67 (−9.34 to 4.00)	0.337
IPAQ (for each point) (Ref Cat No)	0001 (0.001–0.002)	**< 0.001**	0.001 (0.001–0.001)	**< 0.001**
PSQI Total (for each point)	−2.51 (−2.73 to −2.28)	**< 0.001**	−1.57 (−1.79 to −1.35)	**< 0.001**
Impact prolapse symptoms (for each point)	−0.57 (−0.62 to −0.51)	**< 0.001**	−0.20 (−0.27 to −0.12)	**< 0.001**
Impact colorectal-anal symptoms (for each point)	−0.54 (−0.60 to −0.49)	**< 0.001**	−0.15 (−0.22 to −0.09)	**< 0.001**
Impact urinary symptoms (for each point)	−0.39 (−0.43 to −0.34)	**< 0.001**	−0.07 (−0.13 to −0.03)	**0.004**

Bold numerical values are statistically significant values.

## Discussion

Pelvic floor disorders and the impact of their symptoms negatively influence the HRQoL of women. Having an age between 30 and 50 years and physical exercise were related to better HRQoL scores. Other factors were negatively related to women's quality of life, such as: a high BMI, and poor sleep quality. Finally, having a musculoskeletal condition, neoplastic pathology, or mental health illness was also negatively associated with the perceived quality of life.

We highlight as strengths of the study, a priori, the study's sample size, and as a novel aspect the inclusion of all pelvic floor disorders that have previously not been collectively included by other authors. On the other hand, possible limitations include the use of questionnaires for data collection as they may result in selection and memory biases. These biases were considered, and to control it, the questionnaire was previously piloted and elaborated in an adapted language that was easy to read and understand at all educational levels. Another limitation of the study is the non-inclusion of non-Spanish speaking women, as it was not pragmatic to use translators for each of the possible languages. In the same way, an attempt was made to avoid confounding bias, both through the selection of the participants, which was based on defined inclusion criteria defined, and the multivariable analysis, including in the model all those variables that could influence the results obtained, such as age, BMI, background, among others. On the other hand, although a clinical evaluation of pelvic floor dysfunction was not carried out through clinical diagnostic means, the instruments used were questionnaires validated (35, 37) in a population similar to that of our study and are widely accepted on an international level as instruments to detect possible pelvic floor dysfunction (41–43). Likewise, all the other instruments used to measure the different parameters have been validated and previously used in a population similar to that of this study (39, 44–46).

Our results detected an association between age and quality of life, as established by other researchers (47–49). It is important to highlight that the distribution of scores related to quality of life between age groups varies; thus, in line with most of the literature, older age ranges were associated with worse quality of life scores. For example, a longitudinal study carried out in Australia on health and wellbeing included data on 16006 women in three age cohorts and showed a reduction in the scores of the physical component and the mental component throughout the life of the women, negatively influencing age in their older age cohort (48).

The negative impact of BMI identified in our study was also found by García Mendizábal et al. Their study in Spain, which included 1298 women between the ages of 18 and 60, found lower scores for HRQoL as BMI increased (50). This association was also identified by other authors (51).

The presence of specific pathologies negatively influences quality of life. Concerning musculoskeletal pathologies, in line with our results, a study with a nationally representative sample of 17550 participants carried out in the United States, where the same evaluation method was used as in our study, affirmed that those who had musculoskeletal conditions had lower HRQoL scores (52). Likewise, neoplastic diseases, as shown by our results and by other authors (53), are negatively associated with HRQoL. This was also identified in a sample of 1078 women diagnosed with breast cancer in a multi-case control study carried out in Spain, which rated their HRQoL as poor (54).

Another variable that was identified as influencing HRQoL was sleep quality. A longitudinal study in the United Kingdom comprising 30594 participants, of whom 20003 were women, stated that a good quality of sleep was directly and positively related to the quality of life of women (55). In our results, the worse the quality of sleep, the lower the quality of life score.

Regarding the physical activity variable, the results of our study found a positive relationship with better perceived health; the greater the physical activity, the higher the quality of life score. These findings coincide with those in existing literature (56, 57). In Poland, an observational analytical study with a sample size of 598 participants (299 women and 299 men) implemented a physical activity program and demonstrated that the women who performed the program subjectively improved their HRQoL (57).

In relation to mental health, a study carried out in the USA (5719 participants, 48.07% of the population women) found that a depressive state influences how individuals perceive their state of health and wellbeing (58), in agreement with our results and those of other authors (59, 60).

In line with some researchers, it was identified that pelvic floor dysfunction influenced HRQoL (13–16). Specifically, a cross-sectional study carried out in Brazil, including 556 women, identified that all types of urinary incontinence influence women's general quality of life (12). Similarly, a study with 732 enrolled women showed that quality of life was substantially affected in patients with fecal incontinence (16), in line with other authors (11).

On the other hand, in relation to pelvic pain, Ahangari, in a systematic review, only found seven articles with different types of research (cross-sectional, community study, etc.) that addressed this disorder. This systematic review found that this problem affects different dimensions of the women's quality of life, as has also happened in our results (61). Finally, coinciding with what we have identified, an observational analytical study made up of 357 women carried out in Bangladesh found an association between uterine prolapse and related factors and HRQoL (13), as was also determined by other authors (10).

It is necessary to consider HRQoL in women with pelvic floor disorders and who experience the impact of their symptoms in order to be able to implement the necessary measures to address it so that these women can have a better perception of their health on a physical, mental and social level. For this reason, providing greater visibility to this problem experienced by many women will make it possible to implement controls and monitoring at all levels to detect these conditions and provide early care with adequate and effective treatment.

## Conclusions

Women with pelvic floor disorders have a worse perceived quality of life in all dimensions, with prolapse symptoms having the biggest impact, and the emotional component being the most affected sub-domain of HRQoL.

## Data availability statement

The original contributions presented in the study are included in the article/supplementary material, further inquiries can be directed to the corresponding author.

## Ethics statement

The studies involving humans were approved by the study received a favorable opinion from the Research Ethics Committee of the province of Jaen, SPCV-0220/0302-N-20. The studies were conducted in accordance with the local legislation and institutional requirements. The participants provided their written informed consent to participate in this study.

## Author contributions

All authors listed have made a substantial, direct, and intellectual contribution to the work and approved it for publication.
